# Development, validation and feasibility of a Patient Satisfaction Questionnaire for evaluating the quality performance of a diagnostic small fibre neuropathy service: A qualitative study

**DOI:** 10.1111/hex.14011

**Published:** 2024-03-19

**Authors:** Margot Geerts, Janneke G. J. Hoeijmakers, Brigitte A. B. Essers, Ingemar S. J. Merkies, Catharina G. Faber, Mariëlle E. J. B. Goossens

**Affiliations:** ^1^ Department of Neurology, School of Mental Health and Neuroscience Maastricht University Medical Center+ Maastricht The Netherlands; ^2^ Department of Clinical Epidemiology and Medical Technology Assessment Maastricht University Medical Center+ Maastricht The Netherlands; ^3^ Department of Neurology Curacao Medical Center J. H. J. Hamelbergweg Willemstad Curaçao; ^4^ Department of Rehabilitation Research, Department of Rehabilitation Medicine, Research School CAPHRI Maastricht University Maastricht The Netherlands; ^5^ Department of Clinical Psychological Sciences, Department of Clinical Psychological Sciences, Experimental Psychology Maastricht University Maastricht The Netherlands

**Keywords:** diagnostic performance, patient‐reported experience measure, quality of care, qualitative data, questionnaire development, small fibre neuropathy

## Abstract

**Introduction and Aim:**

Small fibre neuropathy (SFN) is a peripheral neuropathy, leading to neuropathic pain and autonomic dysfunction. An evidence‐based standardized patient diagnostic SFN service has been implemented in the Netherlands for improving patient‐centred SFN care. However, the quality of care of this diagnostic SFN service has never been assessed from a patient perspective. The aim of this study was to develop and validate an SFN‐Patient Satisfaction Questionnaire (SFN‐PSQ) to measure the quality performance of a standardized diagnostic SFN service.

**Methods:**

A descriptive qualitative study to create the SFN‐PSQ was performed using the (COREQ (Consolidated Criteria for Reporting Qualitative Research) checklist. For item generation and content development, domains and/or items from validated PSQs were selected. The content development and content validity were performed using a Delphi method with SFN expert caregivers with different backgrounds. By using the three‐step‐test method in individual cognitive interviews, the content validity by patients was finalized.

**Results:**

In one online Delphi panel round, the content of the first concept of the SFN‐PSQ was validated, which resulted in the second concept of the SFN‐PSQ. From July 2019 till March 2020, nine patients consented to participate in the individual cognitive interviews. The most significant changes of the new questionnaire were adding domains and items concerning the waiting list, the diagnostic services and consultation by the hospital psychiatrist. Also, a differentiation was made for both an inpatient and outpatient diagnostic SFN service. Furthermore, the clarity and intelligibility of the domains/items were improved, resulting in an increased comprehension of the SFN‐PSQ. Ultimately, the new developed SFN‐PSQ consisted of 10 domains and 51 items, suitable for measuring patient satisfaction of the neurological analysis in patients with SFN.

**Conclusion:**

Through item generation, expert opinions and interviews with patients, the SFN‐PSQ was developed and validated, and feasibility was confirmed. The structure of the questionnaire, based on the logistic and diagnostic SFN pathway, could be used as a model in other hospitals to improve the quality, continuity and access of SFN care and other chronic diseases taking into account potential cross‐cultural differences.

**Patient or Public Contribution:**

Caregivers were involved in the item generation and content development of the questionnaire. Patients were directly involved in testing the content validity and feasibility of the SFN‐PSQ.

**Clinical Trial Registration:**

Not applicable.

## 1 INTRODUCTION

As a peripheral sensory neuropathy, small fibre neuropathy (SFN) is a neuromuscular disorder.^1^ SFN affects the thinly myelinated and unmyelinated nerve fibres, leading to severe neuropathic pain and autonomic dysfunction.^1^, ^2^ SFN interferes with daily and physical functioning, and thereby contributes to a reduced quality of life.^3^ The prevalence rate of SFN is 53/100,000 inhabitants,^4^ but due to underdiagnosing SFN as a result of difficulties in recognizing SFN symptoms, its incidence and prevalence may be underestimated.^5^ Currently, Dutch hospitals have not implemented a standardized and integrated diagnostic SFN service. Not all diagnostic costs (i.e., skin biopsy) are reimbursed by Dutch health insurance, resulting in use of expensive infrastructure, an increase of patients' burden and substantial health‐related costs. Implementation of a standardized, integrated SFN patient journey is necessary for potentially cost‐saving and efficient, accurate diagnosis of SFN. In addition, it provides a basis for etiological work‐up and treatment decisions. SFN interferes with daily and physical functioning, and thereby contributes to a reduced quality of life.^3^ International SFN referral centres have worked in recent decades, and in close collaboration with the SFN Center in the Netherlands, on improving patient‐centred care (i.e., assessing normative values for intraepidermal nerve fibre density, updating the temperature threshold testing protocol and determining SFN outcome measures by the Rasch methodology) by developing and implementing an interinstitute validated and well‐defined diagnostic SFN approach.^1^, ^6^, ^7^, ^8^ Recently, two studies examined the accessibility and patient satisfaction of occupational therapy services for patients with hereditary transthyretin amyloidosis,^9^ and teleneurology clinics for patients with polyneuropathy,^10^ but no earlier research on patient satisfaction of the interinstitute validated and well‐defined diagnostic SFN approach has been performed.

In 2009, an evidence‐based standardized diagnostic SFN service was implemented at the SFN Center of the Maastricht University Medical Center+ in Maastricht (Maastricht UMC+), the Netherlands. The Department of Neurology is an independent specialty of the Brain and Nerve Center, which includes five other medical specialties, namely, Neurosurgery, Psychiatry, Psychology, Clinical Neurophysiology and Otorhinolaryngology.

The diagnostic SFN service includes a neurological analysis with reserved slots for interview, examination and diagnostic tests at the Neurology Day Care Unit (NDCU) or the outpatient clinic (OC). Yearly, 500 new patients are neurologically analysed at the diagnostic SFN service, and the majority visit the NDCU. Only patients who are living near the hospital visit the OC.

When test results are available, patients are discussed in the multidisciplinary team with all potential stakeholders (neurologists, pain specialists, psychiatrists, psychologists, geneticists, nurse practitioner [NPs], nurses, physiatrists) for patient‐centred advices and treatment options based on evidence‐based practice. As part of the diagnostic SFN service, anxiety and depression are assessed using the Hospital Anxiety and Depression Scale (HADS) questionnaire, which consists of two subscales: one measuring anxiety (HADS‐A) and the other measuring depression (HADS‐D).^11^ In case of a HADS score of ≥14, a patient consultation by the psychiatrist is planned.

Patient satisfaction is considered to be a major indicator in the evaluation and improvement of quality in health care.^12^, ^13^, ^14^, ^15^ Themes of patient satisfaction identified in the healthcare context are provider attitude, technical competence, accessibility and efficacy.^16^ To monitor quality performance from the perspective of the patient, patient surveys are generally accepted tools, provided that the results are combined with qualitative and organizational data.^17^, ^18^, ^19^, ^20^ There are various instruments for measuring patient satisfaction in patients with chronic pain,^21^, ^22^, ^23^ but these instruments often measure the quality of medical care and pain treatment goals, instead of patient satisfaction on the diagnostic process. One validated and feasible core questionnaire for the assessment of patient satisfaction (Core Questionnaire for the assessment of Patient Satisfaction Questionnaire [COPS]) in academic hospitals in the Netherlands has been developed,^24^ with the aim of comparing benchmarking patient satisfaction results among the hospitals. Furthermore, since 2009, the Dutch Federation of University Medical Centers (NFU) has been using the Consumer Quality Index Questionnaire Outpatient Hospital Care (CQI‐QOHC) for measuring customer experiences in hospital admissions.^25^ However, all these Patient Satisfaction Questionnaires (PSQs) are designed to measure the overall quality of general hospital and medical care, but do not cover domains of specific care pathways components, including diagnostic services.

Hence, to improve the quality of SFN care by measuring the patient experience and satisfaction related to the diagnostic SFN service, development of a questionnaire encompassing all relevant aspects of the service is needed. The aim of this study was to develop and validate an SFN‐PSQ for evaluating the quality performance of the diagnostic SFN service from the patients' perspective.

## 2 METHODS

### 2.1 Study design

This study consisted of two phases. The first phase included a descriptive qualitative Delphi study with item generation for the content development and expert opinion of patient satisfaction related to the diagnostic SFN service. The second phase consisted of individual cognitive interviews with patients to test the content validity of the questionnaire. The study was performed at the Maastricht UMC+ in Maastricht, the Netherlands, and covered a period from February 2019 to July 2020.

For creating the content of the SFN‐PSQ, the Dutch guideline for qualitative methods was used.^26^ As suggested in this tool, the expert opinion (i.e., using a Delphi Method), literature, existing PSQs and other additional sources were used to create items that reflect the concept of patient satisfaction of the diagnostic SFN service. Furthermore, individual cognitive interviews were performed for the content validation of the SFN‐PSQ.^27^ The subsequent steps of the content development of the SFN‐PSQ are explained in detail in the text below.

#### 2.1.1 Phase 1a: Item generation and content development of the SFN questionnaire

In the first phase, three existing PSQs (the COPS, CQI‐QOHC and the Patient Assessment of Chronic Illness Care (PACIC) questionnaires)^21^, ^22^, ^24^ and a number of relevant items of a locally developed PSQ of the endoscopy department at the Maastricht UMC+ were selected for creating the conceptual content of the SFN‐PSQ.

The COPS is a reliable questionnaire (*α* = .70–0.91) and consists of six relevant domains and 54 questions for disease‐ and treatment‐related elements of care^24^: (1) Hospital admittance, (2) Nursing care, (3) Medical care, (4) Information, (5) Autonomy and (6) Discharge, with 5‐point Likert‐type answering satisfaction categories (1: *unsatisfied* to 5: *very satisfied*), and a patient characteristic on the general health status (with a 5‐point Likert‐type answering category (1: *bad* to 5: *excellent*).^24^

The CQI‐QOHC questionnaire is based on eight important domains of the OC and/or function departments: (1) Hospital accessibility, (2) Welcome, (3) Facilities, (4) Welcome and stay, (5) Respect and treatment by the doctor, (6) Communication, (7) Going home/discharge and (8) Overall quality assessment, consisting of 55 questions with different answer options (nine closed questions, nine multiple‐choice questions, one open‐ended question and 36 questions with a 4–5‐point Likert scale).^25^ The CQI‐QOHC has good reliability (*α* > .70), except for hospital accessibility (*α* = .64).^25^

The PACIC questionnaire, based on the Chronic Care Model, has five predefined domains: (1) Patient activation, (2) Delivery system/practice design, (3) Goal‐setting/tailoring, (4) Problem‐solving/contextual and (5) Follow‐up/coordination.^28^ The PACIC is a 20‐item questionnaire, which used a 5‐point response scale (from 1: *almost never* to 5: *almost always*).^22^ The domains of the PACIC have reliable Cronbach's *α* (from .71 to .83).^28^

The PSQ from the endoscopy department of the Maastricht UMC+ based on the CQI‐QOHC questionnaire consists of seven domains: (1) Prior to Your Visit, (2) Welcome, (3) Respect and Treatment by the Doctor, (4) Respect and Treatment by the Nurse, (5) Course of the examination, (6) Follow‐up Services with 23 questions with a 4‐point Likert scale (from 1: *no, not at all* to 4: *yes, completely*, and an extra answer option: *not applicable*) and (7) Overall Quality Assessment with a 1‐to‐10 rating scale.

The first step was to select the domains that reflected the multidimensional concept of patient satisfaction with the logistic process of the SFN service (Table 1).

**Table 1:** Content selection of the four Patient Satisfaction Questionnaires for the first concept of the SFN‐PSQ.

SFN‐PSQ	COPS	PSQ endoscopy	CQI Questionnaire Outpatient Hospital Care	PACIC
Domain A: Prior to Your Stay/Waiting List Period				
Q1 information	Q14 (information)			
Q2 accessibility			Q4 (accessibility)	
Q3 accessibility			Q5 (accessibility)	
Q4			Q6 (accessibility)	
Domain B: Welcome and stay at NDCU				
Q5 welcome	Q7 (welcome)			
Q6 welcome			Q8 (welcome)	
Q7 welcome/stay: privacy			Q16 (welcome/stay: privacy)	
Q8 welcome	Q7 (welcome)			
Q9 treatment			Q33 (treatment)	
Q10 treatment	Q11 (treatment)			
Q11 treatment	Q10 (treatment)			
Q12 information	Q14 (information)			
Q13 autonomy	Q18 (autonomy)			
Q14 facilities			Q14 (facilities)	
Q15 waiting time			Q12 (waiting time)	
Q16 waiting time			Q13 (waiting time)	
Domain C: Additional tests (ECG/nerve tests/chest X‐ray)				
Q17 welcome	Q7 (welcome)			
Q18 information/communication	Q15 (information)		Q24 (information/communication)	
Q19 communication			Q21 (communication)	
Q20 welcome/stay: privacy			Q16 (welcome/stay: privacy)	
Q21 course of the examination		QF2 (course of the examination)		
Q22 course of the examination		QF3 (course of the examination)		
Q23 course of the examination		QF4 (course of the examination)		
Q24 information	Q16 (information)			
Domain D: Respect and Treatment by the Doctor or NP				
Q25 treatment/provider attitude			Q19 (treatment/provider attitude)	
Q26 treatment/provider attitude	Q12 (treatment/provider attitude)			
Q27 treatment/provider attitude			Q21 (treatment/provider attitude)	
Q28 treatment/provider attitude			Q22 (treatment/provider attitude)	
Q29 problem‐solving/contextual				Q15 (problem‐solving/contextual)
Q30 goal‐setting/tailoring				Q7 (goal‐setting/tailoring)
Q31 patient activation				Q1 (patient activation)
Q32 goal‐setting/tailoring				Q11 (goal‐setting/tailoring)
Q33 patient activation				Q3 (patient activation)
Q34 autonomy/privacy	Q20 (autonomy/privacy)			
Q35 information	Q15 (information)			
Q36 problem‐solving/contextual				Q12 (problem‐solving/contextual)
Domain E: Respect and Treatment by the Hospital Psychiatry Care Provider				
Q37 treatment/provider attitude			Q19 (treatment/provider attitude)	
Q38 treatment/provider attitude	Q12 (treatment/provider attitude)			
Q39 information/communication			Q28 (information and communication)	
Q40 treatment/provider attitude			Q22 (treatment/provider attitude)	
Q41 information/communication			Q35 (information/communication)	
Q42 information	Q15 (information)			
Q43 information/communication			Q29 (information/communication)	
Domain F: Going Home/Discharge				
Q44 course of the test		QF6 (course of the test)		
Q45 discharge		QH1 (discharge)		
Q46 discharge	Q23 (discharge)			
Q47 discharge	Q22 (discharge)			
Q48 information	Q17 (information)			
Q49 discharge	Q21 (discharge)			
Domain G: Overall Quality Assessment				
Q50 overall quality assessment		QI8 (overall quality assessment)		
Q51 general health status	Q29 (general health status)			

Abbreviations: COPS, Core Questionnaire for the assessment of Patient Satisfaction Questionnaire; CQI, Consumer Quality Index; ECG, electrocardiogram; MD, medical doctor; NCS, nerve conduction studies; NDCU, neurology day care unit; NP, nurse practitioner; PACIC, Patient Assessment of Chronic Illness Care questionnaire; PSQ, Patient Satisfaction Questionnaire; Q, question; SFN, small fibre neuropathy.

#### 2.1.2 Phase 1b: Content validity and feasibility of the SFN questionnaire by the SFN expert team

Subsequently, the content validity and feasibility of the SFN‐PSQ were further developed using a Delphi method with an expert SFN team.^29^, ^30^ This team consisted of three neurologists, two medical doctors, two NPs and three administrative and/or logistic assistants. A Delphi method is a method for obtaining and clarifying group judgements and provides a validated scientific method for resolving complex problems through expert consensus.^31^, ^32^ The SFN‐PSQ was sent by email to all team members for correction and emendation regarding the patient satisfaction content and logistic process of the diagnostic SFN service. Assessment of the SFN‐PSQ was conducted by scoring (yes/no) on understandable language, relevance and redundancy for each question. The SFN expert team could also add additional relevant questions and could give qualitative comments on each domain.

#### 2.1.3 Phase 2: Testing the content validity of the second concept of the SFN‐PSQ by patients

The content validity of the second concept of the SFN‐PSQ was tested using the three‐step test‐interview (TSTI) method^33^ with individual cognitive interviews. A basic definition of cognitive interviewing is administering draft survey questions while collecting additional verbal information about the survey responses, which is used to evaluate the quality of the response or to help determine whether the question is generating the information that its author intends.^34^, ^35^

The target was a sample size of 12 individual cognitive interviews to reach data saturation, as this has been recommended for qualitative studies.^36^, ^37^, ^38^ The sampling method of key informants (based on sex, age, education level and hospital psychiatry visit) was used to select a variety of patients.^39^, ^40^ Patients 18 years of age and older, who completed the neurological analysis of the SFN diagnostic service in the last 6 months (on behalf of the recall period) and were living nearby the region of the Maastricht UMC+, were asked by phone to participate in an individual cognitive interview at the hospital for validating the second concept of the SFN‐PSQ. The duration of the individual cognitive interview was approximately 60–75 min. The audio recordings were typed out by a research assistant and the content was analysed and categorized by one researcher (M. G.).

### 2.2 Study procedure of the individual cognitive interviews of phase 2

With the first patient, a pilot interview was performed by two researchers (M. G. and M. E. J. B. G.) to test the interview procedure, interview guide and interview probes. Data from this pilot interview were also used in the analysis. All other consecutive interviews were performed by one researcher (M. G.), trained in interview skills. All patients were interviewed and audio‐recorded once for single use for the study, and the interviewer's impressions of the interview process were documented by observation notes during the interviews. The three steps of the TSTI method^41^ include an observation of response behaviour^42^ and simultaneous verbalization (e.g., think‐aloud), then exploring the formulations about the items and domains of the questionnaire that are difficult to understand (e.g., probing) and finally obtaining the thoughts and opinions of the patients.^41^

Think‐aloud when completing a questionnaire is unusual and requires a well‐founded explanation and some practice to elicit sufficient think‐aloud behaviour.^33^ Therefore, as a warmup exercise, patients were asked to read aloud the first item (e.g., the date of the neurological analysis) or element (e.g., questionnaire instructions) in the SFN‐PSQ and express any thoughts that came to their mind. To obtain more in‐depth information about the thought process, probing questions were asked while the patient was thinking aloud and completing the SFN‐PSQ. A probing question example for identification of response problems in concordance with the TSTI method is: ‘Can you suggest any changes that would improve the interpretation of the questions in the SFN‐PSQ?’ Within each step of the TSTI, the SFN‐PSQ was adjusted according to the patient's comments, which resulted in a third and final version of the SFN‐PSQ.

### 2.3 Data analysis

The COREQ (Consolidated Criteria for Reporting Qualitative Research) checklist was used to report the study.^43^ The results of the Delphi round were analysed, compared and discussed until consensus was reached by two researchers (M. G. and M.G.O.). Data collection and analysis of the individual cognitive interviews were continued until analytical data saturation was reached.^40^, ^44^ A code system according to a classification by Willis and Lessler,^45^ based on the Question Appraisal System, was used to classify the problems in answering the SFN‐PSQ. In this method, the identified problems of the SFN‐PSQ are classified by the interviewer/researcher into one or more of the following seven categories: Clarity, Knowledge, Assumptions, Response Categories, Sensitively, Instructions and Formatting.^45^ Items and improvements that emerged during the sequential analysis were added to the SFN‐PSQ and verified in the subsequent interviews. It was specifically examined whether the improvements solved the previously found problems when completing the SFN‐PSQ.

Descriptive statistics were used to describe the sociodemographic and logistic features as well as the responses on question level the SFN‐PSQ.

## 3 RESULTS

### 3.1 Phase 1: Item generation, content development and feasibility

Seven domains were chosen for creating the first concept of the SFN‐PSQ: (A) Prior to Your Stay at the NDCU/Waiting List Period, (B) Welcome and Stay at the NDCU, (C) Additional tests ((electrocardiogram [ECG]/nerve tests (nerve conduction studies)/chest x‐ray)), (D) Respect and Treatment from the Doctor or NP, (E) Respect and Treatment by the Hospital Psychiatry Care Provider, (F) Going Home/Discharge and (G) Overall Quality Assessment. These seven domains included in total 51 items scored on a 4‐point Likert scale. This scale included (1) ‘no, not at all’, (2) ‘a little bit’, (3) ‘mostly’ and (4) ‘yes, completely’ and an extra answer option ‘not applicable’. Comment spaces at the end of the themes were created for optional additional feedback.

Table 1 presents the selection of the domains and items of the four PSQs. Autonomy as a separate domain (as in the COPS) was not considered suitable for a 1‐day stay setting. However, two questions of the Autonomy domain were integrated into the SFN‐PSQ domains: (A) Prior to Your Stay at the NDCU/Waiting List Period and (B) Reception and Stay at the NDCU. The third question about the patient's ability to participate in treatment was replaced by another question from the PACIC (Patient activation: Asked about the patient's ideas when making a treatment plan).

### 3.2 Questionnaire adjustments with a Delphi method

From 5 April to 29 April 2019, seven out of 10 expert team members (i.e., three neurologists, two medical doctors and two NPs) who were substantively involved in the SFN diagnostic service responded and discussed the content and feasibility of the SFN‐PSQ. Three administrative and/or logistic assistants did not respond without specific reasons. The SFN expert team made a number of adjustments to the first concept SFN‐PSQ, shown in Figure 1. In summary, the corrections included more specific domains to measure patient satisfaction, grammatical changes to provide more clarity and deletion of items that showed overlap with other items or were not applicable. The results of this Delphi round were integrated into a second concept of the SFN‐PSQ. In the final phase, the content validity and feasibility of the second concept SFN‐PSQ were tested by patients.

Figure 1Content analysis of the first concept of the Small Fibre Neuropathy (SFN)‐Patient Satisfaction Questionnaire by the SFN expert team. NDCU, neurology day care unit; Q, question; SFN‐PSQ‐2, Small Fibre Neuropathy‐Patient Satisfaction Questionnaire, version 2.

**Figure:**
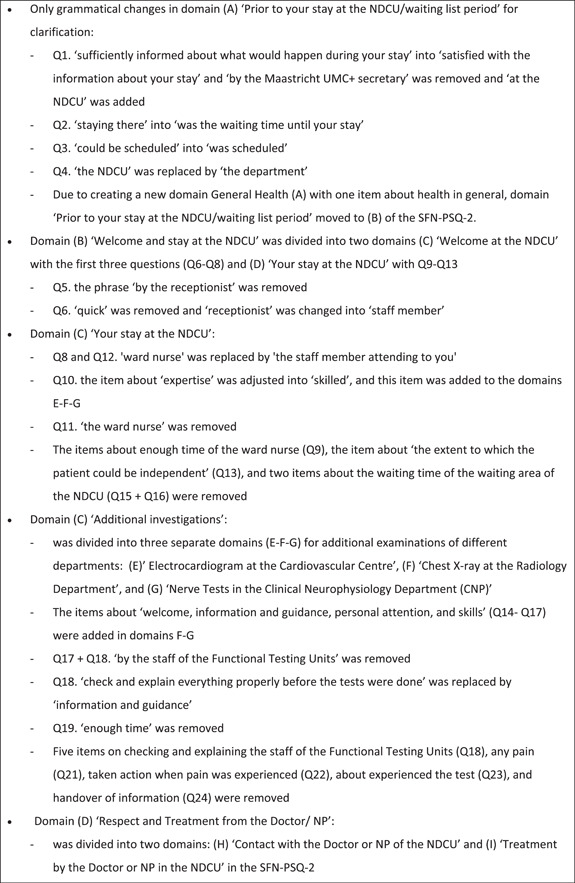

**Figure:**
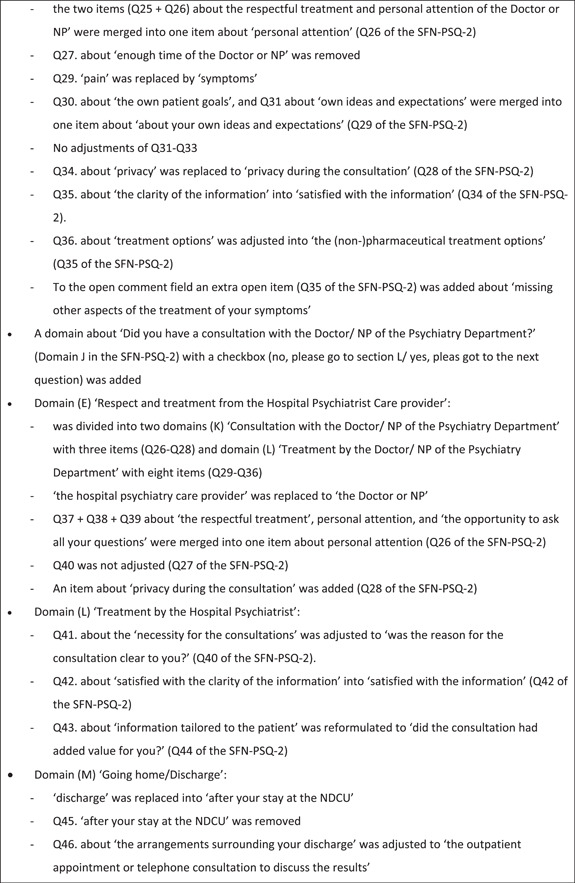

**Figure:**
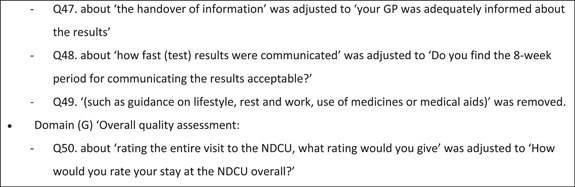

### 3.3 Content validity by individual cognitive interviews with patients (phase 2)

From July 2019 till March 2020, nine consecutive patients participated for testing the content validity and feasibility of the second concept of the SFN‐PSQ. Table 2 presents the logistic features and demographics of the patients. In eight of the nine patients, the diagnosis of SFN was confirmed. Average age was 46.1 (range: 26.0–62.0) years, and five patients were female. The level of education of patients was taken into account, with approximately equal numbers of participating patients with high and medium levels of education.

**Table 2:** Characteristics of the study population of individual cognitive interviews.

	*N* = 9
Age (years)	
Average (CI)	46.1 (26.0–62.0)
Median (IQR)	52.5 (42.0–53.0)
Sex (*n*, %)	
Female	5 (55.6)
Level of education	
Low	0 (0.0)
Medium	5 (55.6)
High	4 (44.4)
Diagnosis of SFN (*n*, %)	8 (88.9)
Diagnostic tests of SFN (*n*, %)	
Normal skin biopsy and normal temperature threshold testing	1 (11.1)
Abnormal skin biopsy and abnormal temperature threshold testing	6 (66.7)
Abnormal skin biopsy	2 (22.2)
Abnormal temperature threshold testing	0 (0.0)
SFN diagnostic service at the 1‐day stay visit at the NDCU or OC (*n*, %)	
One‐day stay NDCU	8 (88.9)
One‐way travel distance (km)	
Average (CI)	91.4 (67.7–81.2)
Median (IQR)	74.5 (44.8–95.6)
Period of visit at the SFN diagnostic service	September 2018–October 2019
General health status (*n*, %)	
Bad	0 (0.0)
Moderate	4 (44.4)
Good	5 (55.6)
Very good	0 (0.0)
HADS, median (IQR)	
Anxiety	6.6 (4.9–8.3)
Depression	6.4 (4.3–8.5)
Time duration of completing the SFN‐PSQ, min (IQR)	14 (11–20)

Abbreviations: CI, confidence interval; HADS, Hospital Anxiety and Depression Scale; IQR, interquartile range; NDCU, neurology day care unit; OC, outpatient clinic; SFN, small fibre neuropathy; SFN‐PSQ, Small Fibre Neuropathy‐Patient Satisfaction Questionnaire.

Neurological analysis at the NDCU occurred in eight patients; in one patient, the neurological analysis was carried out at the OC. General health status was rated as good in five patients. The median scores of anxiety and depression symptoms were 6.6 (confidence interval [CI]: 4.9–8.3) for anxiety and 6.4 (CI: 4.3–8.5) for depression. The average duration for completing the SFN‐PSQ was 14 min (IQR: 11–20).

Figure 2 shows the iterative process for adjusting the SFN‐PSQ by the individual cognitive interviews.

Figure 2Iterative process for adjusting the SFN‐PSQ by individual cognitive interviews. MD, medical doctor; NDCU, neurology day care unit; NP, nurse practitioner; SFN, small fibre neuropathy.

**Figure:**
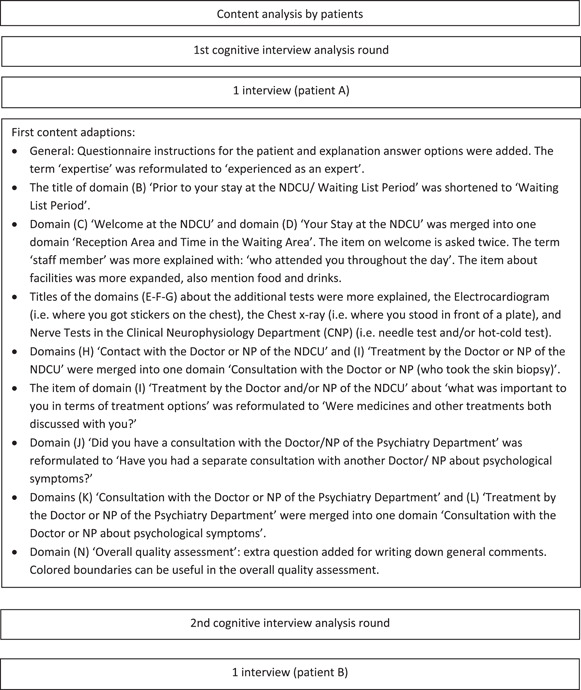

**Figure:**
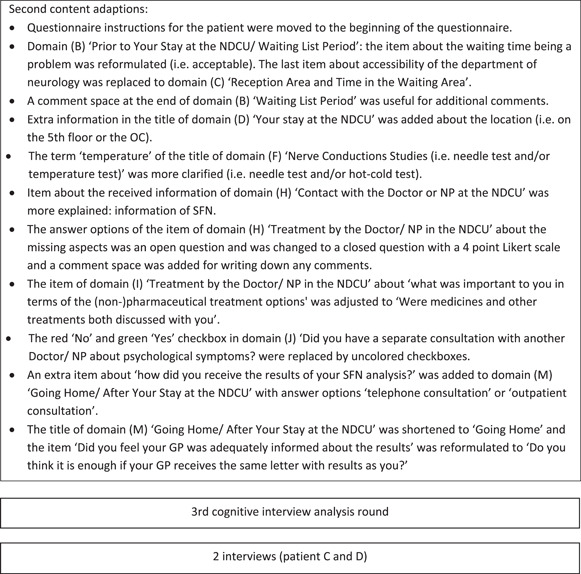

**Figure:**
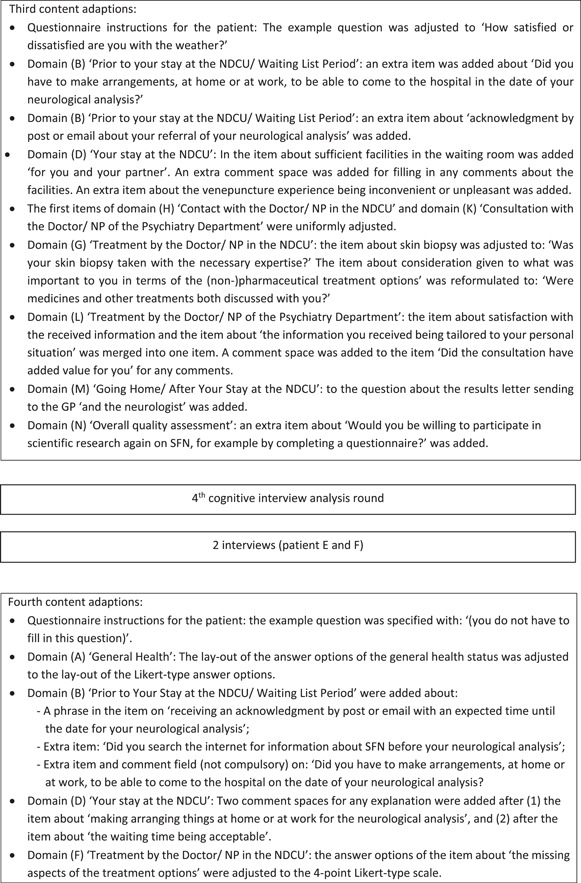

**Figure:**
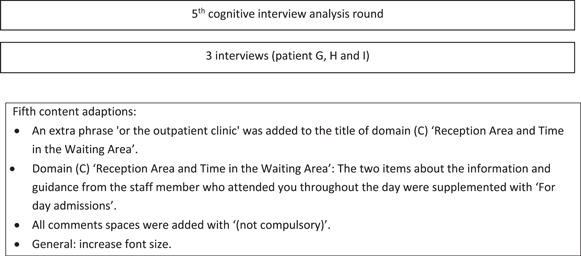

After the first interview, the SFN‐PSQ was modified and presented in the interview with patient B. The results of the following two interviews in the third analysis round were combined and implemented, and the adjusted SFN‐PSQ was presented to patients E and F. The final and third draft of the SFN‐PSQ was created after the fourth analysis round of the last three patients (G, H and I). The most important changes to the SFN‐PSQ questionnaire were addition of domains and items concerning the Prior to your Stay at the NDCU/Waiting List Period, the diagnostic services and consultation with the Doctor/NP of the Psychiatry Department. Furthermore, a differentiation was made for the logistic feature of the diagnostic SFN service (i.e., the NDCU or the OC), which was integrated into the final SFN‐PSQ.

### 3.4 Changes in the final questionnaire

Table 3 presents a summary of the adapted domains, items and answer options of the second concept of the SFN‐PSQ by the individual cognitive interviews, as a solution for the identified bottlenecks. Most questionnaire problems were classified into clarity, response categories, formatting and instructions. No questionnaire problems were found in the category knowledge, sensitivity or assumptions. Because of low diagnostic yield, the use of routine ECG was stopped during the study. Therefore, domain (D) ECG was removed from the third and final concept of the SFN‐PSQ (Appendix S1), which consisted of 10 domains with 51 items.

**Table 3:** Categorization of problems of the cognitive individual interviews.

	Interview patient A	Interview patient B	Interview patient C and D	Interviews patient E and F	Interviews patient G, H and I
*Clarity*: Problems with the intent of meaning of a question. Subcategories: Wording, technical term, vague, lack of reference period	Domain (C) ‘Welcome at the NDCU’: The term ‘staff member’ was more explained with: ‘who attended you throughout the day’. The item about facilities need to be more expanded, also mention food and drinks. More explanation is needed for the titles of the domains (E–F–G) Electrocardiogram (i.e., where you got stickers on the chest), the chest X‐ray (i.e., where you stand in front of a plate), and Nerve Tests in the CNP (i.e., needle test and/or hot–cold test). Title of domain (H) ‘Contact with the Doctor or NP of the NDCU’ was more clarified with ‘(e.g., who took the skin biopsy)’. The item of domain (I) ‘Treatment by the Doctor and/or NP of the NDCU’ about the values and traditions in the recommended treatment options had to be reformulated to was reformulated into ‘Were medicines and other treatments both discussed with you?’ Domain (J) was reformulated to ‘Did you have a separate consultation with another Doctor/NP about psychological symptoms?’	Domain (B) ‘Prior to Your Stay at the NDCU/Waiting List Period’: The item about the waiting time being a problem was reformulated (i.e., acceptable). Extra information in the title of domain (C) ‘Welcome at the NDCU’ was added about the location (i.e., on the fifth floor). The term ‘temperature’ of the title of domain (F) ‘Nerve Conductions Studies (i.e., needle test and/or temperature test)’ was clarified more (i.e., needle test and/or hot–cold test). Item about the information provided of domain ((I) ‘Treatment by the Doctor/NP in the NDCU’ was more explained: information of SFN. The item of domain (I) ‘Treatment by the Doctor/NP in the NDCU’ about ‘what was important to you in terms of the (non‐)pharmaceutical treatment options’ was adjusted to ‘Were medicines and other treatments both discussed with you’. The title of domain (M) ‘Going Home/After Your Stay at the NDCU’ was shortened to ‘Going Home’ and the item ‘Did you feel your GP was adequately informed about the results’ was reformulated to ‘Do you think it is enough if your GP receives the same letter with results as you?’	Domain (C) ‘Reception Area and Time in the Waiting Area’: In the item about sufficient facilities was added ‘for you and your partner’. Domain (G) ‘Treatment by the Doctor/NP in the NDCU’: The item about skin biopsy was adjusted to: ‘Was the skin biopsy with the necessary expertise?’ The item about consideration given to what was important to you in terms of the (non‐)pharmaceutical treatment options' was reformulated to: ‘Were medicines and other treatments both discussed with you?’ Domain ((M) ‘Going Home/After Your Stay at the NDCU’: to the question about the results letter sending to the GP ‘and the neurologist’ was added.	Extra items of domain (B) ‘Prior to Your Stay at the NDCU/Waiting List Period’ were added about: A phrase in the item on ‘receiving an acknowledgment of receipt by post or email with an expected time until the date for your neurological analysis’; Extra item: ‘Did you search the internet for information about SFN before your neurological analysis’; Extra item and comment field (not compulsory) on: ‘Did you have to make arrangements, at home or at work, to be able to come to the hospital on the date of your neurological analysis?	An extra phrase ‘at the outpatient clinic’ was added to the title of domain (C) ‘Reception Area and Time in the Waiting Area’. Domain (C) ‘Reception Area and Time in the Waiting Area’: The two items about the information and guidance from the staff member ‘who attended you throughout the day’ were supplemented with ‘For day admissions’.
*Response categories*: Problems with the response categories. Subcategories: Missing, mismatch, question–answer, vague, open‐ended questions, overlapping, illogical order	Domain (N) ‘Overall quality assessment’: Extra question added for writing down general comments. Coloured boundaries can be useful in the overall quality assessment.	A comment space at the end of domain (B) ‘Prior to Your Stay at the NDCU/Waiting List Period’ was useful for additional comments. The item of domain (H) ‘Treatment by the Doctor/NP in the NDCU’ about the missing aspects of the treatment was an open question and was changed to a closed question with a 4‐point Likert scale and a comment space was added for writing down any comments. The red ‘No’ and green ‘Yes’ checkbox of domain (J) ‘Did you have a separate consultation with another Doctor/NP about psychological symptoms?’ were replaced by uncoloured checkboxes. Domain (M) ‘Going Home/After Your Stay at the NDCU’: An extra item about ‘how did you receive the results of your SFN analysis?’ was added with answer options ‘telephone consultation’ or ‘an outpatient clinic visit’.	Domain (B) ‘Prior to your stay at the NDCU/Waiting List Period’: An extra item was added about ‘Did you have to make arrangements, at home or at work, to be able to come to the hospital in the date of your neurological analysis?’ Domain (B) ‘Prior to your stay at the NDCU/Waiting List Period’: An extra item about ‘acknowledgment by post or email about your referral of your neurological analysis’ was added. Domain (D) ‘Your stay at the NDCU’: An extra comment space was added for filling in any comments about the facilities. Domain (C) ‘Reception Area and Time in the Waiting Area’: An extra item about the venepuncture experience being inconvenient or unpleasant was added. Domain (L) ‘Treatment by the Doctor/NP of the Psychiatry Department’: A comment space was added to the item ‘Did the consultation have added value for you’ for any comments. Domain (M) ‘Going Home/After Your Stay at the NDCU’: An extra item about ‘Would you be willing to participate in scientific research again on SFN, for example by completing a questionnaire?’ was added.	Domain (D) ‘Your stay at the NDCU’: Two comment spaces for any explanation were added after (1) the item about arranging things at home or at work for the neurological analysis and (2) after the item about the waiting time being acceptable. Domain (F) ‘Treatment by the Doctor/NP in the NDCU’: The answer options of the item about ‘the missing aspects of the treatment options’ were adjusted to the 4‐point Likert‐type scale.	
*Instructions*: Problems with introductions, instructions of explanations	General: Questionnaire instructions for the patient and explanation answer options were added. The term ‘expertise’ was reformulated to ‘experienced as an expert’.		Questionnaire instructions for the patient: The example question was adjusted to ‘How satisfied or dissatisfied are you with the weather?’	Questionnaire instructions for the patient: The example question was specified with: ‘(you don't have to fill in this question)’.	All comments spaces were added with ‘(not compulsory)’.
*Formatting*: Problems with layout or question ordering	The title of domain (B) ‘Prior to Your Stay at the NDCU/Waiting List Period’ was shortened to ‘Waiting list period’. Domain (C) ‘Welcome at the NDCU’ and domain (D) ‘Your Stay at the NDCU’ were merged into one domain ‘Reception Area and Time in the Waiting Area’. Domain (C) ‘Welcome at the NDCU’ and domain (D) ‘Your Stay at the NDCU’: The item on welcome is asked twice. Domains (H) ‘Contact with the Doctor or NP of the NDCU’ and (I) “Treatment by the Doctor or NP of the NDCU’ were merged into one domain ‘Consultation with the Doctor or NP (who took the skin biopsy)’.	Questionnaire instructions for the patient were moved to the beginning of the questionnaire. The last item about accessibility of the department of neurology was replaced to domain (B) ‘Prior to Your Stay at the NDCU/Waiting List Period’ was replaced to domain (C) ‘Reception Area and Time in the Waiting Area’.	The first items of domain (H) ‘Contact with the Doctor/NP in the NDCU’ and domain (K) ‘Consultation with the Doctor/NP of the Psychiatry Department’ were uniformly adjusted. Domain (L) ‘Treatment by the Doctor/NP of the Psychiatry Department’: The item about satisfaction with the received information and the item about ‘the information you received being tailored to your personal situation’ was merged into one item.	‐ Domain (A) ‘General Health Status’: The layout of the answer options of the general health status was adjusted to the layout of the Likert‐type answer options.	General: Increase font size.

Abbreviations: CNP, Clinical Neurophysiology Department; ECG, electrocardiography; MD, medical doctor; NCS, nerve conduction studies; NDCU, neurology day care unit; NP, nurse practitioner; SFN, small fibre neuropathy.

## 4 DISCUSSION

We developed a new questionnaire, the SFN‐PSQ, encompassing all relevant aspects related to patient satisfaction of the diagnostic SFN service of the Maastricht UMC+.

Although several existing PSQs can be used for measuring patient satisfaction of patients with chronic pain, a questionnaire from the patient's perspective covering the logistic and diagnostic pathway of a SFN service, including Prior to your stay/Waiting List Period, diagnostic services and a consultation with the Hospital Psychiatry Care Provider, was lacking. By selecting relevant domains/items (e.g., Welcome and Stay, Information, Respect and Treatment, Discharge) of the most suitable PSQs, the first concept of the SFN‐PSQ was created.

During the Delphi round, the expert SFN caregivers achieved uniformity in the items about personal attention, expertise and privacy in one‐on‐one patient contacts.

After the input of the experts, the content validity of the SFN‐PSQ was tested in a sample of patients with SFN‐related complaints in daily practice. The most significant changes were adding domains and items concerning the waiting list period, diagnostic services and a consultation by the Hospital Psychiatry Care Provider. Also, a differentiation was made for both inpatient and outpatient neurologic analysis, ensuring that the SFN‐PSQ is applicable in all patients. Furthermore, the individual cognitive interviews improved the clarity and intelligibility of the items, which has increased the comprehension of the SFN‐PSQ from the patients' perspective. Ultimately, this resulted in a feasible and reliable SFN‐PSQ consisting of ten domains with 51 items, with a shorter average completion time than previously expected (approximately 20 min). This has shown that the final SFN‐PSQ is suitable for measuring patient satisfaction in all patients who are visiting hospitals, where a similar diagnostic SFN service has been set up.

The strength of the study is that the content development of the SFN‐PSQ was carried out in close collaboration with expert SFN caregivers with different backgrounds, but above all, the considerable influence of the patients on the content validity and user‐friendliness of the SFN‐PSQ.

Another strength is that the content validity of the questionnaire with experts and individual cognitive patient interviews confirmed that the SFN‐PSQ is relevant and comprehensive.

A potential limitation is that the development and content validity of the SFN‐PSQ were performed in a single‐centre study and were not tested in centres outside the Netherlands. As there is only one diagnostic SFN service in the Netherlands, some domains and/or items of the SFN‐PSQ may not be applicable as they are not consistent with the diagnostic SFN service in other countries. In addition, to ensure that translation is appropriate for the target group,^46^, ^47^ a linguistic validation procedure for the three SFN‐PSQs has been performed.

Another limitation is that perhaps a small sample of mainly Caucasian expert SFN caregivers (85.7%) and patients (100%) participated in this study. However, the study was conducted according to the qualitative research guidelines and the interviews continued until data saturation was achieved. In addition, the SFN experts were from different professional backgrounds, ensuring that the content of the questionnaire was evaluated from different perspectives.

Second, although the number of individual cognitive patient interviews was small, the heterogeneity (sex, age, education, HADS scores) of the patients included is consistent with the general SFN population,^8^, ^48^ and the results may therefore be generalized to the general SFN population. Nevertheless, measurement of the cross‐cultural validity of the SFN‐PSQ is recommended in a future study with a mixed‐ethnicity study population.^49^ After the cross‐cultural validation process of the SFN‐PSQ, the questionnaire could be used as a model for other chronic conditions, such as Parkinson's disease or epilepsy, but also in developing care pathways for other medical specialties.

## 5 CONCLUSION

The content validity and feasibility of the SFN‐PSQ were developed and validated through item generation, expert opinions and interviews by patients. For future use of the SFN‐PSQ, its construction based on the logistic and diagnostic SFN pathway could be a blueprint for other hospitals to measure patient satisfaction to optimize the quality of the logistic and diagnostic SFN pathway, other chronic diseases and care pathways from patients' perspective.

## AUTHOR CONTRIBUTIONS

**Margot Geerts**: Conceptualization; investigation; writing—original draft; formal analysis; project administration; methodology; validation; writing—review and editing; data curation. **Janneke G. J. Hoeijmakers**: Conceptualization; writing—original draft; methodology; validation; writing—review and editing; supervision; formal analysis; resources. **Brigitte A. B. Essers**: Writing—original draft; writing—review and editing; validation; methodology; supervision; formal analysis. **Ingemar S. J. Merkies**: Conceptualization; writing—original draft; writing—review and editing; methodology; validation; formal analysis; supervision; resources. **Catharina G. Faber**: Conceptualization; writing—original draft; methodology; writing—review and editing; validation; formal analysis; supervision. **Mariëlle E. J. B. Goossens**: Data curation; supervision; resources; formal analysis; writing—review and editing; validation; methodology; conceptualization; investigation; writing—original draft.

## CONFLICT OF INTEREST STATEMENT

Janneke G. J. Hoeijmakers reports a grant from the Prinses Beatrix Spierfonds (W.OK17‐09), outside the submitted work. Catharina G. Faber reports grants from the European Union's Horizon 2020 research and innovation programme. Marie Sklodowska‐Curie grant for PAIN‐Net, Molecule‐to‐man pain network (grant no. 721841), grants from Prinses Beatrix Spierfonds, grants from Grifols and Lamepro for a trial on IVIg in small fibre neuropathy, other from Steering committees/advisory board for studies in small fibre neuropathy of Biogen/Convergence and Vertex, outside the submitted work. Ingemar S. J. Merkies reports grants and nonfinancial support from Grifols, grants from Lamepro, during the conduct of the study; other from participation in steering committees of the Talecris ICE Study, CSL Behring, LFB, Novartis, Octapharma, Biotest and UCB, outside the submitted work. The remaining authors declare no conflict of interest.

## ETHICS STATEMENT

The study protocol was not subjected to the Medical Research Involving Human Subjects Act (WMO), which was confirmed by the Medical Ethics Committee of Maastricht UMC+ (2019‐1049). Informed consent was obtained before participating in the study, according to the principles of the Declaration of Helsinki.^50^

## Supporting information

Supporting information.

## Data Availability

The data that support the findings of this study are available on request from the corresponding author. All data relevant to the study are included in the article. The data are not publicly available due to privacy or ethical restrictions.
