# Supplementary material for: Development, validation and feasibility of a Patient Satisfaction Questionnaire for evaluating the quality performance of a diagnostic small fibre neuropathy service: A qualitative study
Source: Health Expect. 2024 Mar 19;27(2):e14011. doi: 10.1111/hex.14011 (PMC10951422; doi:10.1111/hex.14011)
Supplement: Supplementary file 1 — Supporting information. [file HEX-27-e14011-s001.pdf]

Maastricht UMC+

Patient Satisfaction Survey

Department of Neurology **Small Fibre Neuropathy Centre of Expertise**

QUESTIONNAIRE COMPLETION DATE ☐ - ☐ - ☐ (day-month-year, e.g. 10-06-2018)

INSTRUCTIONS FOR THE PATIENT

- Answer all the questions by ticking the box; you can select only one answer.
- Completing the questionnaire will take you about 20 minutes.
- There are no 'right' or 'wrong' answers.
- The questions below are about your stay at the Neurology Day Care Unit. Please read each question carefully and indicate what you think is the most appropriate answer at the time of completing the questionnaire.
- You can use the explanation about the answer options below to help you answer the questions. Sample question: How satisfied or dissatisfied are you with the weather? (you do not have to fill in this question)

no, not at all	a little	mostly	yes, completely

A. GENERAL HEALTH

		poor	fair	good	very good	excellent
1	**In general, how do you feel about your overall health?**	❑	❑	❑	❑	❑

	B. WAITING LIST PERIOD	no, not at all	a little	mostly	yes, completely	not applicable
2	Did you receive an acknowledgement by post or email about your referral, with an expected time until the date of your neurological analysis?	❑	❑	❑	❑	❑
3	How satisfied were you with the information about the neurological analysis that came with the acknowledgement?	❑	❑	❑	❑	❑
4	Did you search the internet for information about small fibre neuropathy before your neurological analysis?	❑	❑	❑	❑	❑
5	Did you have to make arrangements, at home or at work, to be able to come to the hospital on the date of your neurological analysis? Please use the field below for any comments (not compulsory).	❑	❑	❑	❑	❑

Comments:

6	When you received the appointment confirmation and the questionnaire by post or email, did you receive an internet link for additional information about small fibre neuropathy, for example for the website: www.dvnexpertisecentrum.nl?	❑	❑	❑	❑	❑
7	Did you find the waiting time until the neurological analysis acceptable? Please use the field below for any comments (not compulsory).	❑	❑	❑	❑	❑

Comments:

	C. RECEPTION AREA AND TIME IN THE WAITING AREA (ON THE 5th FLOOR OR THE OUTPATIENT CLINIC)	no, not at all	a little	mostly	yes, completely	not applicable
8	Were you able to find the department or outpatient clinic easily in the hospital?	❑	❑	❑	❑	❑
9	Was the receptionist helpful?	❑	❑	❑	❑	❑
10	Did the front desk provide enough privacy?	❑	❑	❑	❑	❑
11	For day admissions: were you satisfied with the welcome by the staff member who attended to you throughout the day?	❑	❑	❑	❑	❑
12	Did you find the staff member skilled at drawing blood?	❑	❑	❑	❑	❑

	C. RECEPTION AREA AND TIME IN THE WAITING AREA (ON THE 5th FLOOR OR THE OUTPATIENT CLINIC)	no, not at all	a little	mostly	yes, completely	not applicable
13	Did you find the blood test inconvenient or unpleasant?	❑	❑	❑	❑	❑
14	Were you given enough personal attention?	❑	❑	❑	❑	❑
15	For day admissions: were you satisfied with the information and guidance from the staff member who attended to you throughout the day?	❑	❑	❑	❑	❑
16	Were there adequate facilities in the waiting room of the outpatient clinic for you and your partner (including magazines, food and drinks)? Please use the field below for any comments (not compulsory).	❑	❑	❑	❑	❑

Comments:

	D. CHEST X-RAY (WHERE YOU STOOD IN FRONT OF A PLATE)	no, not at all	a little	mostly	yes, completely	not applicable
17	Were you satisfied with how you were welcomed by the staff member?	❑	❑	❑	❑	❑
18	Were you satisfied with the information and guidance provided by the staff member?	❑	❑	❑	❑	❑
19	Were you given enough personal attention?	❑	❑	❑	❑	❑
20	Did you consider the staff member performing the chest x-ray to have the necessary expertise?	❑	❑	❑	❑	❑

	E. NERVE TESTS (NEEDLE TEST AND/OR HOT-COLD TEST)	no, not at all	a little	mostly	yes, completely	not applicable
21	Were you satisfied with how you were welcomed by the staff member?	❑	❑	❑	❑	❑
22	Were you satisfied with the information and guidance provided by the staff member?	❑	❑	❑	❑	❑
23	Were you given enough personal attention?	❑	❑	❑	❑	❑
24	Did you consider the doctor performing the test(s) to have the necessary expertise?	❑	❑	❑	❑	❑

	F. CONSULTATION WITH THE DOCTOR/NURSE PRACTITIONER (WHO TOOK THE SKIN BIOPSY)	no, not at all	a little	mostly	yes, completely	not applicable
25	Were you given enough personal attention?	❑	❑	❑	❑	❑
26	Did you consider the doctor/nurse practitioner to have the necessary expertise?	❑	❑	❑	❑	❑
27	Was there enough privacy during the consultation?	❑	❑	❑	❑	❑
28	Were you asked about your own ideas, expectations or experiences?	❑	❑	❑	❑	❑
29	Were you asked about the impact of your symptoms on your daily life?	❑	❑	❑	❑	❑
30	Were you asked about your lifestyle (including smoking, exercise, diet)?	❑	❑	❑	❑	❑
31	Were you asked about your experiences with medicines and their effects or side effects?	❑	❑	❑	❑	❑
32	Were you satisfied with the information you received about small fibre neuropathy? Please use the field below for any comments (not compulsory).	❑	❑	❑	❑	❑

Comments:

33	Was your skin biopsy taken with the necessary expertise?	❑	❑	❑	❑	❑
34	Were medicines and other treatments both discussed with you?	❑	❑	❑	❑	❑
35	Did you miss other aspects of the treatment of your symptoms? If yes, could you indicate which aspects you missed?	❑	❑	❑	❑	❑

Comments:

G. HAVE YOU HAD A SEPARATE CONSULTATION WITH ANOTHER DOCTOR/NURSE PRACTITIONER ABOUT PSYCHOLOGICAL SYMPTOMS?	NO please go to I (going home)	YES please go to H (consultation about psychological symptoms)
	❑	❑

	H. CONSULTATION WITH THE DOCTOR/NURSE PRACTITIONER ABOUT PSYCHOLOGICAL SYMPTOMS	no, not at all	a little	mostly	yes, completely	not applicable
36	Were you given enough personal attention?	❑	❑	❑	❑	❑
37	Was there enough privacy during the consultation?	❑	❑	❑	❑	❑
38	Did you consider the doctor/nurse practitioner to have the necessary expertise?	❑	❑	❑	❑	❑
39	Was the reason for the consultation clear to you?	❑	❑	❑	❑	❑
40	Was the information you received tailored to your personal situation?	❑	❑	❑	❑	❑
41	Did the consultation have added value for you? Please use the field below for any comments (not compulsory).	❑	❑	❑	❑	❑

Comments:

	I. GOING HOME	no, not at all	a little	mostly	yes, completely	not applicable
42	Did you go home satisfied?	❑	❑	❑	❑	❑
43	Did you know whom to contact with questions or problems?	❑	❑	❑	❑	❑

		telephone consultation	outpatient consultation
44	How did you receive the results of your small fibre neuropathy analysis?	❑	❑

		no, not at all	a little	mostly	yes, completely	not applicable
45	Were you satisfied with how your results were discussed?	❑	❑	❑	❑	❑
46	Do you find the 8-week period for receiving the results acceptable?	❑	❑	❑	❑	❑
47	Do you think it is enough if your GP and neurologist receive the same letter with results as you?	❑	❑	❑	❑	❑
48	Were you satisfied with the advice and/or information on further treatment? Please use the field below for any comments (not compulsory).	❑	❑	❑	❑	❑

Comments:

J. GENERAL

49 Tick the box to indicate your satisfaction with the neurological analysis as a whole:

1	2	3		4	5	6	7	8		9	10
no, not at all			a little			mostly			yes, completely		

		yes	no
50	Would you be willing to participate in scientific research again on small fibre neuropathy, for example by completing a questionnaire?	❑	❑

51 If you have any other general comments, please note them here:

Comments:

Thank you very much for completing this questionnaire.
